# Structure–Activity Relationship of Synthetic Cathinones: An Updated Review

**DOI:** 10.1021/acsptsci.4c00299

**Published:** 2024-08-06

**Authors:** Núria Nadal-Gratacós, Martalu D. Pazos, David Pubill, Jorge Camarasa, Elena Escubedo, Xavier Berzosa, Raúl López-Arnau

**Affiliations:** † Department of Pharmacology, Toxicology and Therapeutic Chemistry, Pharmacology Section and Institute of Biomedicine (IBUB), Faculty of Pharmacy, University of Barcelona, 08028 Barcelona, Spain; ‡ Chemical Reactions for Innovative Solutions (CRISOL), IQS School of Engineering, Universitat Ramon Llull, 08017 Barcelona, Spain

**Keywords:** synthetic cathinones, new psychoactive substances, structure−activity relationship, reward, psychostimulant, cytotoxicity

## Abstract

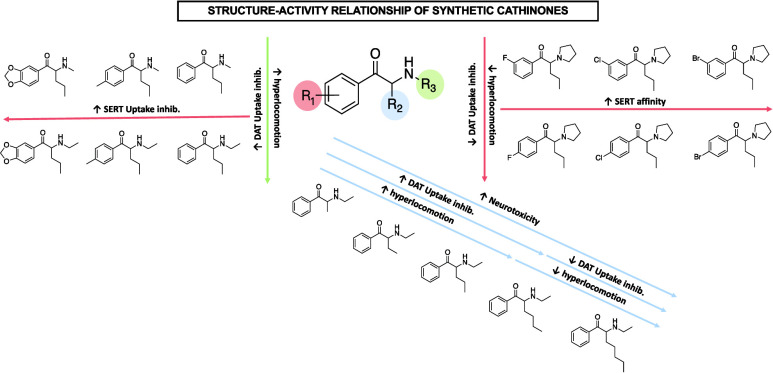

The escalating prevalence of new psychoactive substances (NPSs) poses a significant public health challenge, evidenced by the vast chemical diversity, with over 500 substances reported annually to the United Nations Office on Drugs and Crime-Early Warning Advisory (UNODC-EWA) in the past five years. Among NPSs, synthetic cathinones are gaining a lot of popularity among users. Notably, synthetic cathinones accounted for approximately 50% of the total quantity of NPSs reported as seized by EU Member States in 2021. Preliminary data from UNODC indicates that a total of 209 synthetic cathinones have been reported to date. As their popularity grows, studying the structure–activity relationship (SAR) of synthetic cathinones is essential. SAR studies elucidate how structural features impact biological effects, aiding in toxicity prediction, regulatory compliance, and forensic identification. Additionally, SAR studies play a pivotal role in guiding drug policies, aiding authorities in categorizing and regulating newly emerging synthetic cathinones, mitigate public health risks and offer valuable insights into potential therapeutic applications. Thus, our Review consolidates recent findings on the effects of different substitutions in the chemical scaffold of synthetic cathinones on their mechanism of action as well as pharmacological and toxicological effects of synthetic cathinones, thus enhancing understanding of the SAR of synthetic cathinones’ pharmacology and potential implications.

The global illicit drug market has been characterized in the last decades by the emergence of New or Novel Psychoactive Substances (NPS). NPS are substances of abuse specifically designed to mimic the effects of already prohibited drugs. The term “new”, does not necessarily refer to new inventions but to substances that have recently become available.^1^ According to the United Nations Office on Drugs and Crime (UNODC), NPS can be classified into six groups based on their mode of action: stimulants, synthetic cannabinoid receptor agonists, dissociatives, sedatives/hypnotics, synthetic opioids and classic hallucinogens.^2^ The proliferation of NPS in the illicit market presents a significant Public Health concern due to the rapid increase in their chemical diversity. The European Union (EU) Early Warning System (EWS), established in 1997, was the first regional early warning system for monitoring NPS and the UNODC-Early Warning Advisory (EWA), established in 2013, was the first global monitoring system for NPS monitoring. Both systems have significantly contributed to the surveillance and assessment of NPS prevalence worldwide. Since monitoring started in 1997 until December 2022, a total of 930 NPS have been reported to EU-EWS, 41 of which were first reported in 2022.^3^ Globally, between December 2021 and May 2023, a total of 1228 NPS have been reported to the UNODC-EWA, 44 of which were first reported in 2022.^2^ The variability in the number of reports per country highlights that the challenges they face differ drastically from country to country. The number of reports of new NPS detected per year has decreased since 2016, reflecting the efforts made to control and restrict the sale of NPS.^4^ However, the annual count of reported substances has remained consistent, with over 500 substances being reported to UNODC-EWA in each of the last 5 years.^5^ Stimulants and synthetic cannabinoid receptor agonists constitute the two largest groups of NPS worldwide accounting 66% of reported NPS.^5^ Preliminary data from UNODC indicates that a total of 209 synthetic cathinones have been reported to date.^6^ Notably, synthetic cathinones accounted for approximately 50% of the total quantity of NPS reported as seized by EU Member States in 2021.^3^ More precisely, the latest data underscores that the seizures of NPS in Europe were primarily driven by three synthetic cathinones −3-chloromethcathinone (3-CMC), 3-methylmethcathinone (3-MMC) and 4-chloromethcathinone (4-CMC)-, accounting for 48% of all NPS seizures and highlighting their potential to take a significant role in Europe’s stimulant market.^3^ These substances are ß-ketone amphetamine compounds derived from cathinone, an active alkaloid naturally occurring in the khat shrub (*Catha edulis*). The khat shrub is commonly grown in East Africa and Southern Arabia, where it is not uncommon to find people chewing its leaves or brewing them in tea for their mild stimulant effects.^7,8^ The effects that they produce are characterized by stimulant, empathogenic and euphoric properties, similar to those of amphetamine.^9^ Synthetic cathinones are commonly found as white, yellowish or brown crystal-like powder and they are typically consumed through swallowing, injection, snorting, or smoking. These substances are colloquially known as “bath salts” and are often labeled as “not for human consumption”, “research chemicals” or “for external use only” to circumvent legal restrictions.^10^

The emergence of the first synthetic cathinones can be traced back to the mid-20th century. The first synthetic analog of cathinone, methcathinone (MCAT), was synthesized by Roger Adams’s lab in the early 1920s, and later patented by Parke-Davis as an analeptic agent.^11,12^ However, due to its stimulant properties, it soon found its way into the realm of recreational drug use. Reports of MCAT abuse began to surface in the late 1980s–early 1990s, particularly in Eastern Europe and Russia.^13,14^ Mephedrone (4-methylmethcathinone; 4-MMC) and 3,4-methylenedioxypyrovalerone (MDPV) were described for the first time in 1929 and in 1969, respectively, for research purposes.^15,16^ In the early 21st century, both 4-MMC and MDPV made significant breakthroughs in the designer drug market and both gained rapid popularity.^17−19^ 4-MMC users have described that acute administration of this cathinone results in increased energy, euphoria, talkativeness and empathy, although adverse effects are also commonly reported such as excessive sweating, tachycardia, anxiety, forgetfulness and tiredness among others.^20^ The introduction of these first synthetic cathinones into the recreational drug market brought about a shift in drug use patterns. Users sought novel experiences and alternatives to traditional substances, and synthetic cathinones provided a legal and easily accessible option.^21^ Governments around the world responded by implementing emergency scheduling and enacting legislation to control and regulate synthetic cathinones.^22^ In response to legal restrictions, clandestine chemists modified the chemical structures of synthetic cathinones, leading to the emergence of new analogs. This process of structural modification allowed manufacturers to circumvent existing laws and regulations, making it difficult for authorities to keep pace with the evolving landscape of synthetic cathinones.

Our review offers novel and essential insights into the Structure–Activity Relationship (SAR) of synthetic cathinones, filling a crucial gap in existing literature. While numerous reviews have delved into the predominant psychostimulant, addictive, and neurotoxic effects of synthetic cathinones emerging in the illicit drug market,^10,23−25^ they often neglect to thoroughly investigate the correlation between these effects and their chemical structure. Given the proliferation of new cathinones in the illicit drug market, our review capitalizes on this opportunity to delve deeper into understanding the SAR of these compounds, offering valuable insights that advance our comprehension of their pharmacological properties and potential implications.

## Mechanism of Action

Synthetic cathinones exert their effects by interfering with the regular functioning of monoamine transporters (MATs), whose primary function is to terminate monoamine signaling by taking up the respective substrate from the synaptic cleft into the neuronal cytoplasm. MATs are single polypeptide chains that contain approximately 600 amino acids with 12 transmembrane helices, with intracellular N- and C-termini, connected with flexible intracellular and extracellular loops, that are found within the presynaptic neuronal terminals of their respective pathways.^26,27^ These transporters belong to the solute carrier 6 (SLC6) family and operate as secondary active cotransporters. These include the dopamine transporter (DAT; SLC6A3), the serotonin transporter (SERT; SLC6A4, also present in platelets), and the norepinephrine transporter (NET; SLC6A2). They utilize the transmembrane Na+ electrochemical gradient to actively transport their substrates across the cell membrane. MATs are classified as symporters, as they transport Na+ ions in the same direction as their substrate, although some transporters also facilitate the exchange of a K+ ion per substrate, exhibiting antiport activity.^26,28,29^ While DAT and NET facilitate the transport of a single molecule of dopamine (DA) or norepinephrine (NE) along with two Na+ ions and one Cl– ion, SERT operates by moving one serotonin (5-HT) molecule alongside one Na+ ion and one Cl– ion, along with one K+ ion in the opposite direction.^27,30^ Once inside the cell, monoamines can either be degraded or recycled and stored into synaptic vesicles thanks to the vesicular monoamine transporters 1 and 2 (VMAT1 and VMAT2), which utilize a proton gradient to capture cytosolic monoamines.^30^

**Figure 1:**
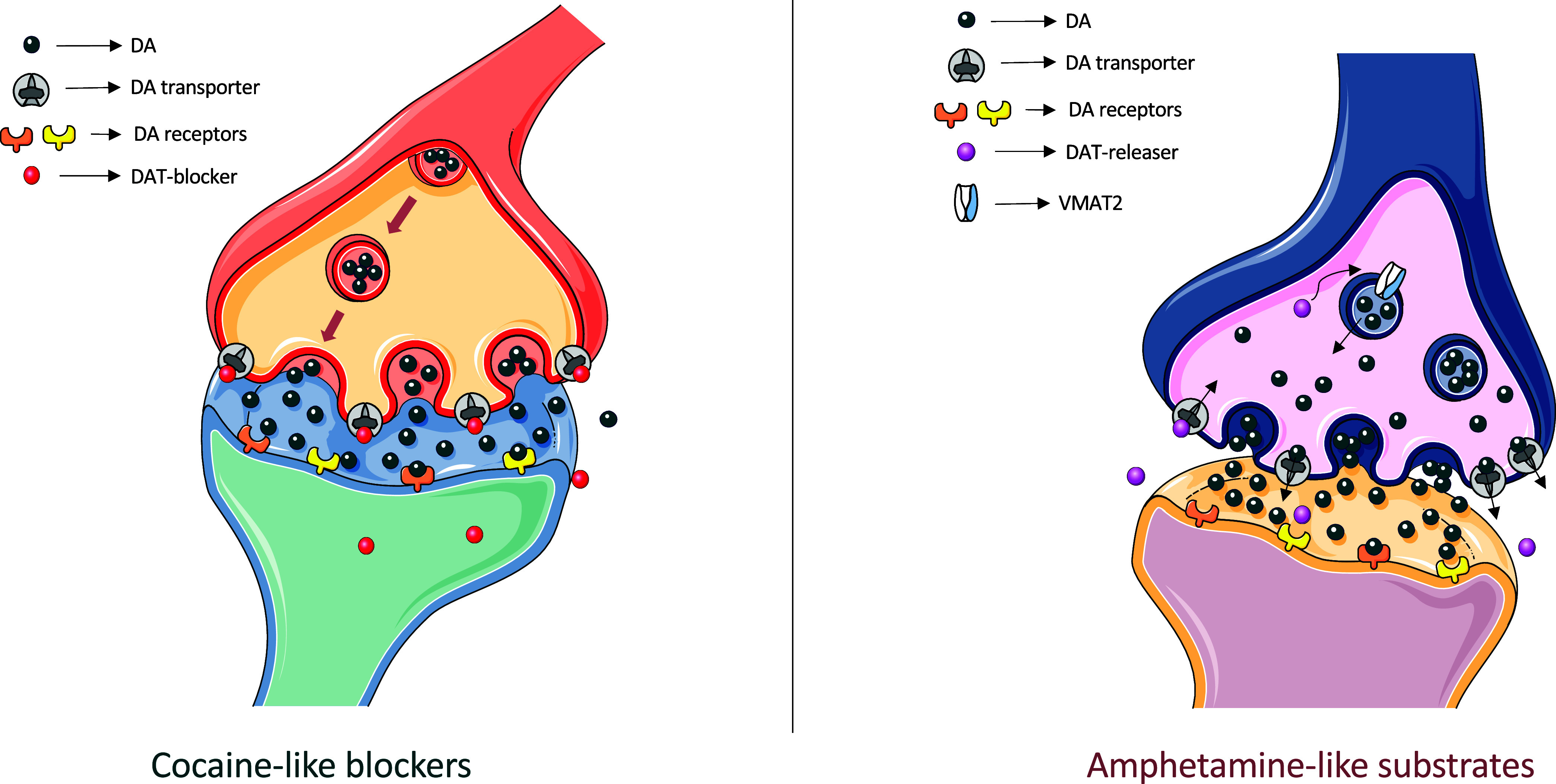
Mechanism of action of cocaine-like blockers and amphetamine-like substrates.

All synthetic cathinones are able to increase extracellular monoamine concentrations in the brain, therefore enhancing cell-to-cell monoamine signaling, although depending on the cathinone this mechanism is triggered slightly different, as they can act as cocaine-like “inhibitors”- therefore binding to the active site of the transporter and blocking uptake of neurotransmitters from the extracellular medium - or amphetamine-like “substrates” – which also bind to the orthosteric site, but are subsequently translocated into the neuronal cytoplasm, where they may interact with the VMAT and impede the filling of synaptic vesicles, hence triggering efflux of intracellular neurotransmitters (see Figure 1).

Cathinone was described to be able to block the uptake and induce release of tritiated DA in synaptosomal preparations. Moreover, the same authors reported that repeated administration of d,l-cathinone produces long-lasting DA depletions but no alterations on the brain levels of NE nor 5-HT.^31^ Similar results were reported in human embryonic kidney (HEK293) cells, in which cathinone was able to induce DA release but no 5-HT release.^32^ Several authors have reported that the first generation synthetic cathinones 4-MMC and methylone can inhibit neurotransmitter uptake and act as substrates at DAT, NET and SERT, both in rat brain synaptosomes and in HEK293 cells.^32−35^ On the other hand, MDPV and its second generation derivatives act mainly as DAT and NET blockers.^36,37^

## Psychostimulant and Rewarding Properties of Synthetic Cathinones

### Psychostimulant Effects

The hyperlocomotion effect of amphetamines is primarily due to dopaminergic activation of the mesolimbic pathway, although the nigrostriatal pathway, which connects the substantia nigra to the striatum in the basal ganglia, is also involved in movement control.^38−42^ In fact, overstimulation of the nigrostriatal pathway can lead to tremors and other movement disorders, which are also seen in chronic methamphetamine users.^43^

As described in the last section, psychostimulants interact with the DAT, NET and SERT, although DAT is the main responsible for the locomotor effects observed after administering these compounds.^44^ In fact, it has been described that administration of cocaine or amphetamine to DAT knockout animals does not result in an increase in locomotion.^45^ On the contrary, mice overexpressing the DAT display an increased amphetamine-induced locomotion.^46^ Furthermore, endogenous 5-HT also plays a role in the locomotor effects of psychostimulants. For instance, López-Arnau et al.^47^ reported that pretreatment with the 5-HT2A receptor antagonist ketanserin resulted in a decrease in the hyperlocomotor effects observed for methylone, butylone and 4-MMC.

Cathinone induces a dose-dependent increase in locomotor activity mediated by dopaminergic neurons in rats, which is significantly higher in females.^48−50^ MCAT has been demonstrated to stimulate locomotion through activation of dopaminergic neurotransmission.^51^ Its close analogs, 4-MMC and methylone showed to be nonselective substrates at MATs in a similar potency to 3,4-methylenedioxymethamphetamine (MDMA), and elicited psychostimulant effects *in vivo*, although in less extent than methamphetamine.^34^ The psychostimulant effects of several second generation synthetic cathinones have also been reported. Gatch and colleagues reported the locomotor effects of a range of doses of pentedrone, pentylone, 3-fluoromethcathinone (3-FMC), and 4-methylethcathinone (4-MEC), with psychostimulant effects lasting up to 6h.^52^ Wojcieszak et al. reported that dopaminergic neurotransmission played an important role in the psychostimulant effects induced by α-pyrrolidinopentiophenone (α-PVP), α-pyrrolidinohexiophenone (α-PHP) and α-pyrrolidinooctanophenone (α-POP).^53^ Moreover, our group recently showed a correlation of the binding efficacy at DAT with an increase in locomotion at an intermediate dose (10 mg/kg) of *N*-etlyl-pentedrone (NEPD) analogs.^54^

### Reward

DA is the primary neurotransmitter associated with reward and pleasure. The assessment and processing of potential rewards are facilitated by a linked network of brain regions known as the brain reward circuitry. The mesolimbic dopaminergic pathway plays a central role in reward processing. It originates primarily in the ventral tegmental area (VTA) of the midbrain and projects to limbic structures, most notably the Nucleus accumbens (NAcc), a nucleus located in the ventral striatum (VS) linked to motivation and reward.^55,56^

Notably, increased DA levels have been observed in response to both natural rewards and the administration of drugs of abuse. For example, microdialysis in the NAcc revealed a 37% rise in extracellular DA when rats pressed a lever for a food reward.^57^ This increase was even more pronounced after cocaine administration.^57^ Similarly, sexually active male rats exhibited a significant increase in DA levels in the NAcc, and to a lesser extent in the dorsal striatum (DS), during copulation.^58^ Human studies administering amphetamine to seven drug-naïve healthy volunteers demonstrated activation of the medial orbitofrontal cortex, the rostral part of the anterior cingulate cortex, and the VS, supporting the idea that the initial use of psychostimulants activates the reward circuitry.^59^ A study published in 1990,^60^ demonstrated an increase of DA levels both in NAcc and anterior caudate-putamen of Sprague–Dawley rats after an acute administration of cathinone. Likewise, an increase of DA levels in the VS has also been reported after administration of second generation synthetic cathinones.^51,61,62^

Numerous studies have associated a high DAT/SERT ratio of psychoactive drugs with an increased risk of abuse liability.^63−65^ Roberts and colleagues^66^ found that the DAT/SERT selectivity ratio served as a superior predictor of self-administration of cocaine analogs compared to the affinity at DAT alone. Furthermore, Negus and Banks^63^ established a correlation between the maximum increase in total intracranial self-stimulation (ICSS) and the selectivity for DAT vs SERT. For example, amphetamine is known to elevate extracellular DA levels, fenfluramine increases 5-HT levels, and MDMA can increase both DA and 5-HT levels in the rat NAcc, as determined by *in vivo* microdialysis.^67,68^ Self-administration studies have revealed that amphetamine exhibits more robust reinforcing effects than MDMA, while fenfluramine does not produce reinforcement.^63,69−71^ These findings suggest that a high DAT/SERT ratio indicates a likelihood for a drug to have reinforcing and rewarding effects. On the other hand, potent DAT and NET inhibition -but not SERT- is correlated with lower pharmacological doses of stimulants, including synthetic cathinones, in humans.^72^

As previously described, synthetic cathinones are able to inhibit monoamine transporters. Several authors have reported that a great variety of cathinones show high DAT/SERT ratios,^36,37,73^ suggesting that these compounds may have abuse potential. The rewarding and reinforcing effects of a variety of synthetic cathinones have been studied and demonstrated *in vivo*. In fact, the reinforcing and rewarding effects of cathinone were described in 1980 and 1990, respectively.^74,75^ MCAT was the first synthetic cathinone to be self-administered in primates.^76^ The rewarding and reinforcing effects of 4-MMC, MDPV and methylone, first generation synthetic cathinones were soon described by different research groups after gaining popularity as recreational drugs.^77−79^ Moreover, a study published in 2018 showed a correlation between the reinforcing effectiveness of pyrrolidine-containing cathinones–determined by progressive ratio - and their selectivity for DAT vs SERT.^80^

## Subjective Effects of Synthetic Cathinones

The effects of synthetic cathinones are individual-, dose- and route of administration-dependent.^81^ They represent a structurally diverse class of compounds that exist along a mechanistic spectrum from amphetamine-like monoamine-releasing agents to cocaine-like uptake inhibitors, with a broad selectivity profile. This is why a diversity in the subjective effects (and side effects) of synthetic cathinone users is also expected, with some resembling the empathogenic effects induced by MDMA (methylone, butylone), while others exhibit stimulant and compulsive effects akin to cocaine consumption (pentylone).^82,83^ The reasons that lead users to choose cathinones instead of cocaine, MDMA, or methamphetamine include the lower price and the wrong perception of safety.^81,84^ It is important to highlight that the final subjective experience will be conditioned by multiple factors including the drug (dose, purity, route, substance, drug combination), the user (age, sex, race, genetic predispositions, individual’s mental and physical condition, sleep, nutrition, history of drug use, interactions, purpose of use, mood) and the setting (environment, company, peer pressure).^85,86^

The primary effects sought by users include euphoria, increased energy and alertness, elevated mood and sense of well-being, increased motivation and empathy, productivity and work capacity, self-confidence, sociability, talkativeness, hallucinations, insomnia, reduced appetite, increased libido, and sexual drive.^83,87,88^ The side effects can be divided into somatic effects, which include tachycardia, hypertension, nausea, vomiting, mydriasis, seizures, hyperthermia, bruxism, nasal irritation, dry mouth, and difficulty urinating; and psychological effects, which include anxiety unpleasant ‘comedown, hallucinations, confusion, anorexia, psychosis, compulsive use and dependence symptoms.^23,81,84^ A web-based survey of 104 recreational users of synthetic cathinones highlighted that 60% of consumers take synthetic cathinones once or more per month, that the most common route of administration was snorting (40%), and that the three most reported drugs used in combination with synthetic cathinones were marijuana (75%), tobacco (73%), and alcohol (72%).^84^ The most prevalent effects included a mixture of pleasant and unpleasant rewards: increased energy (94%), tachycardia (91%), difficulty sleeping (89%), euphoria (88%), loss of appetite (88%), dry mouth (85%), sex drive (79%) and bruxism (79%). The three most reported consequences were tolerance (57%), having neglected one’s responsibilities (56%), and personality change (49%). The most frequently reported acute subjective effects by 70 drug consumers who had tried 4-MMC tablets were euphoria, excitement, and improved mood (90%), craving for the drug (87%), and increased energy (80%). Overall, most users considered the effects enjoyable (83%), compared to the remaining (17%) who reported an unpleasant experience.^84^

However, not all the effects are only related to cathinone use, as polysubstance abuse (for example with alcohol, tobacco, MDMA, cannabis, and cocaine) is very common among those taking synthetic cathinones.^1,84^ In addition, synthetic cathinones are often found in powders or pills resembling one another, potentially leading consumers to be unaware of the specific stimulant or combination of substances they are using. Adulteration by both substitution and addition is particularly relevant in this family of substances, as these mixtures increase the risk for consumers to encounter unforeseen adverse effects and potential harm.^3^ Moreover, the instability of the ecstasy market in EU countries caused an adulteration by partial substitution of MDMA with synthetic cathinones due to their ability to evoke similar effects.^89^ In 2022, an increase in synthetic cathinones (4-CMC, 3-MMC, 4-MMC, and dipentylone) mis-sold as MDMA or used to adulterate it, was reported to the EWS.^3^ Even though they share chemical similarities; they are not necessarily equivalent in respect to the risk they pose to public health. MDMA is known for its distinct subjective effects including improved empathy, sociability, and enhanced appreciation of music attributed to a more selective binding for SERT than DAT. However, in contrast to MDMA, most cathinones such as 4-MMC induce strong feelings of craving in most users.^89,90^ Moreover, 4-MMC users reported a prolonged feeling of activity compared to cocaine.^1,81,84^ Both animal and human studies have also suggested that methylone may possess an addiction potential like or greater than MDMA, although results suggested a lower potential for abuse and compulsive use than cocaine, methamphetamine, and 4-MMC.^42,79,91^ On the other hand, an observational-naturalistic study conducted in humans to evaluate the acute pharmacological effects of methylone oral self-administration, showed that methylone was able to induce the prototypical psychostimulant and empathogenic effects generally associated with MDMA, although with lower intensity.^82^

## Toxicity and Forensic Identification of Synthetic Cathinones

Synthetic cathinones have been linked to a range of severe toxic effects.^23,24^ In fact, the intoxication potential of cathinones is notably high due to their potent psychostimulant properties, which can lead to compulsive redosing and unpredictable behavior; for a review see.^10^ Case report studies involving synthetic cathinones usually report agitation, disorientation, aggression, tachycardia, tachypnea, hyperthermia dyspnea, psychosis and even suicidal behavior.^92−94^ As reported in the European Drug Report 2023, in 2021 3-MMC alone was involved in 68 acute drug toxicity cases in different hospitals in the EU.^3^ Moreover, the lethality associated with cathinone use is alarming, with numerous reported fatalities stemming from both direct toxic effects and risky behaviors induced by intoxication. In fact, drug-induced deaths involving synthetic cathinones almost doubled in 2020 in 7 EU countries.^3^ The major causes of death from synthetic cathinones intoxication include acute kidney failure, cardiovascular complications, multiple organ failure and serotonin syndrome.^94−97^

Regarding forensic identification, presumptive color tests can be used initially to identify which class of compounds a substance belongs to, followed by confirmatory techniques. According to the UNODC, the most suitable presumptive test for synthetic cathinones is the Zimmermann color test (sometimes referred to as the Janovsky test).^98^ Other techniques include gas chromatography coupled with (tandem) mass spectrometry (GC-MS/MS)—widely regarded as the gold-standard method—high-performance liquid chromatography with (tandem) mass spectrometry (HPLC-MS/MS), ion mobility spectrometry (IMS), capillary electrophoresis (CE), and immunoassays.^98,99^ Unfortunately, these techniques have certain disadvantages, such as high costs, complex operations, and lengthy analysis times. Additionally, the identification process is complicated by the vast and constantly evolving array of synthetic cathinones, their often low concentrations in biological samples, and the frequent presence of these substances in mixtures with other compounds.

Identification of metabolites of synthetic cathinones is also a useful tool as they may serve as additional markers of abuse in toxicological analyses.^100^ Moreover, these metabolites can retain some of the parent compound’s psychoactive effects and contribute to the overall pharmacological and toxicological profile of the drug.^101^ Some of the most common metabolic pathways of cathinones are reduction of the keto group, hydroxylation of the alkyl chains and N-dealkylation.^100,102^ The main metabolites of MCAT are ephedrine and norephedrine. Reduction of the carbonyl group to an alcohol decreases NET release activity by about 3-fold when transitioning from methcathinone to (−)-ephedrine. This reduction in activity is slightly more pronounced when the stereochemistry around the benzylic atom is reversed, as in (+)-pseudoephedrine. Additionally, reversing the stereochemistry at the α position of (−)-ephedrine results in nearly a 100-fold decrease in activity, whereas the same stereochemical change in (+)-pseudoephedrine has no impact on activity, as seen in (+)-ephedrine.^101^ This reduction of activity by reduction of the ketone to an alcohol has even a greater negative impact on dopamine release. On the other hand, reduction of the carbonyl group resulted in inactive compounds at the SERT.^101^ SAR studies focusing on active metabolites of synthetic cathinones would further enhance the understanding of the pharmacological profile and potential impacts on public health of these substances.^100,103^

The chemical structure of cathinones also seem to have an important role in determining their main metabolites. For instance, Lopes et al. 2023,^100^ reported that the main metabolic pathway for N-ethyl cathinones such as 4-methyl-α-ethylaminopentiophenone (4-MeAP) and 4-methyl-*N*-ethylbuphedrone (4-MNEB) was hydroxylation of the alkyl chain, while for 4′-methyl-*N*,*N*-dimethylpentedrone (4-MDMP), a NN-dimethyl cathinone, the main metabolic pathway was demethylation followed by hydroxylation of the aliphatic side chain. On the other hand, the main metabolic pathway for chlorinated cathinones seems to be reduction of the keto group. However, for some chlorinated cathinones hydroxylation of the alkyl chain seems to be equally important.^100^

Moreover, many synthetic cathinones are known to be unstable in biological matrices.^104^ Therefore, understanding drug stability in biological samples is also essential for researchers and forensic toxicologists to correctly evaluate analytical data. Glicksberg and Kerrigan^105^ reported that cathinones containing tertiary amines were significantly more stable than those containing secondary amines. In fact, synthetic cathinones with methylenedioxy or N-pyrrolidine ring show higher degradation resistance, and acidification of samples PH increases their stability.^106^ On the other hand, unsubstituted and ring-substituted secondary amines seem to have the least stability, but addition of a methylenedioxy group seems to have a stabilizing effect for cathinones with secondary and tertiary amines.^105,107^

## Therapeutic Potential of Synthetic Cathinones

Chewing khat leaves for its stimulant effects is a widespread practice in some countries, often undertaken to enhance social interaction or improve performance among other reasons.^108^ In fact, khat users in Yemen have reported to believe that khat offers therapeutic benefits for various minor ailments, including headaches, colds, minor body pains, arthritis, fevers and depression,^109^ therefore associating khat consumption with perceived therapeutic effects.

Some synthetic cathinones have been studied for different therapeutic approaches. For instance, diethylpropion was first developed in the early 1960s by the pharmaceutical company Temmler-Weke as an anorectic agent,^110^ although in 2020 the European Medicines Agency (EMA) recommended its withdrawal of marketing authorizations as studies revealed users were taking the drug for long periods of time therefore increasing the risk of important adverse effects.^111^ Another synthetic cathinone, α-PVP, was first synthesized and patented as a central nervous system stimulant and pressor agent in 1963 by Boehringer Ingelheim,^112^ although it never reached the market. A close analog, pyroavalerone, was used as an antifatigue and to treat lethargy, although it was soon discontinued due to its addictive potential.^113,114^ As of now, bupropion is the only synthetic cathinone approved for the Food and Drug Administration (FDA). Bupropion has been described both as an antidepressant and as a smoking cessation agent.^115,116^ Moreover, a combination of dextromethorphan and bupropion (Auvelity) was approved for major depressive disorder treatment in 2022.^117−119^ In this scenario, bupropion is used for its ability to competitively inhibit CYP2D6, therefore increasing dextromethorphan’s bioavailability.^117^

Research on the therapeutic use of synthetic cathinones has been limited on account of the rise in significant concerns due to the potential for adverse effects and abuse of the gran majority of them. Nevertheless, their therapeutic possibilities should not be disregarded. In fact, several authors have suggested that synthetic cathinones may be beneficial for the treatment of depression, obesity, anxiety, muscle spasms, airway diseases, tobacco smoking-cessation and stimulants abuse, among others.^120−125^ Therefore, examining the SAR of synthetic cathinones is crucial, not only for pinpointing compounds with a higher likelihood of abuse potential but also for identifying those substitutions that may lead to cathinones lacking rewarding effects and with fewer toxicological concerns. This dual approach is essential in guiding research toward potential therapeutic candidates among synthetic cathinones, emphasizing the need for a balanced exploration that encompasses both the risks and the therapeutic possibilities inherent in these substances.

## Synthesis

**Figure 2:**
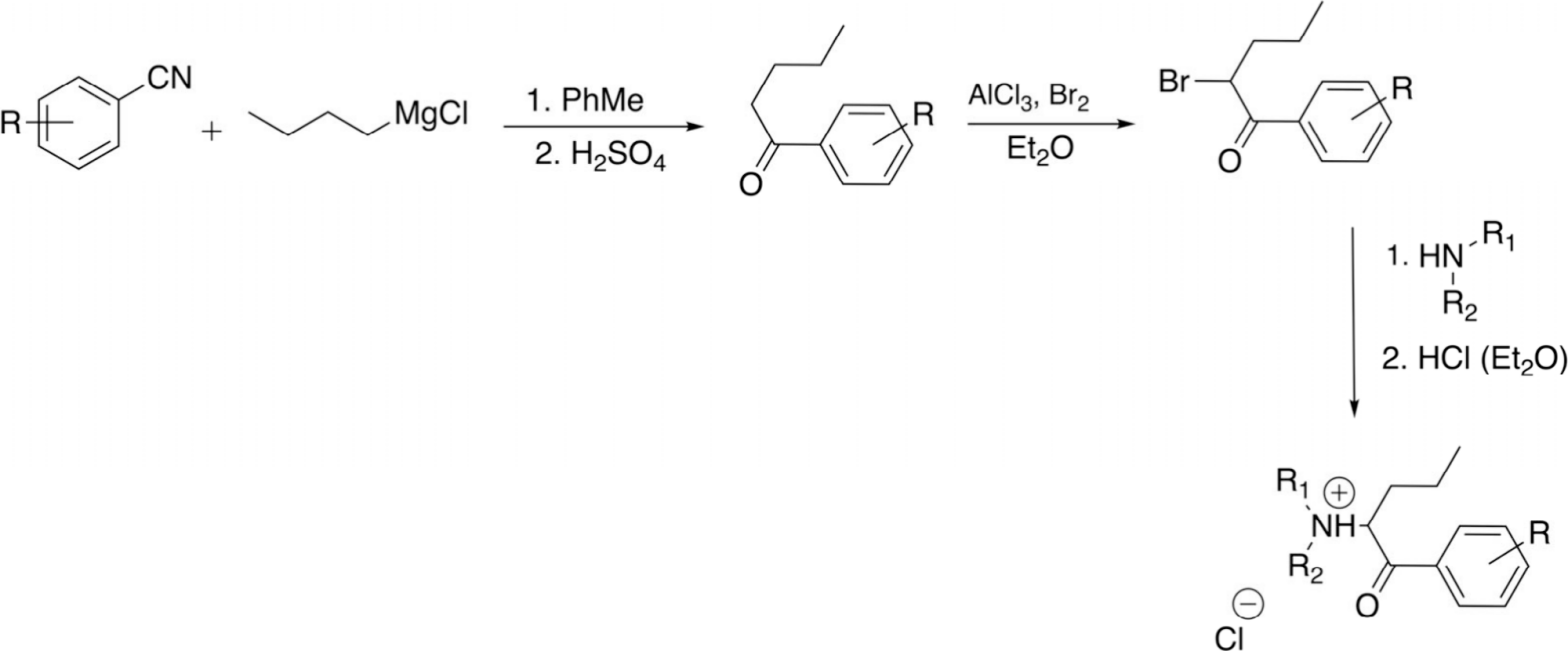
General synthetic route for the obtention of synthetic cathinones.

Arylnitriles (1) are subjected to reaction with a Grignard reagent, followed by acidic hydrolysis (H_2_SO_4_) to synthesize ketones (2). Alternatively, 2 can be synthesized by a Friedel–Crafts acylation. Two are then brominated selectively by addition of bromine in the presence of aluminum trichloride in catalytic amounts to achieve the corresponding α-bromoketone (3), which is then subjected to reaction with the corresponding amine to achieve the desired synthetic cathinone, typically isolated as hydrochloride salt. For more details regarding the synthesis of pyrovalerone analogues, check Meltzer et al. 2006.^126^ The general synthetic route for the obtention of synthetic cathinones is shown in Figure 2.

## Structure–Activity Relationship of Synthetic Cathinones

**Figure 3:**
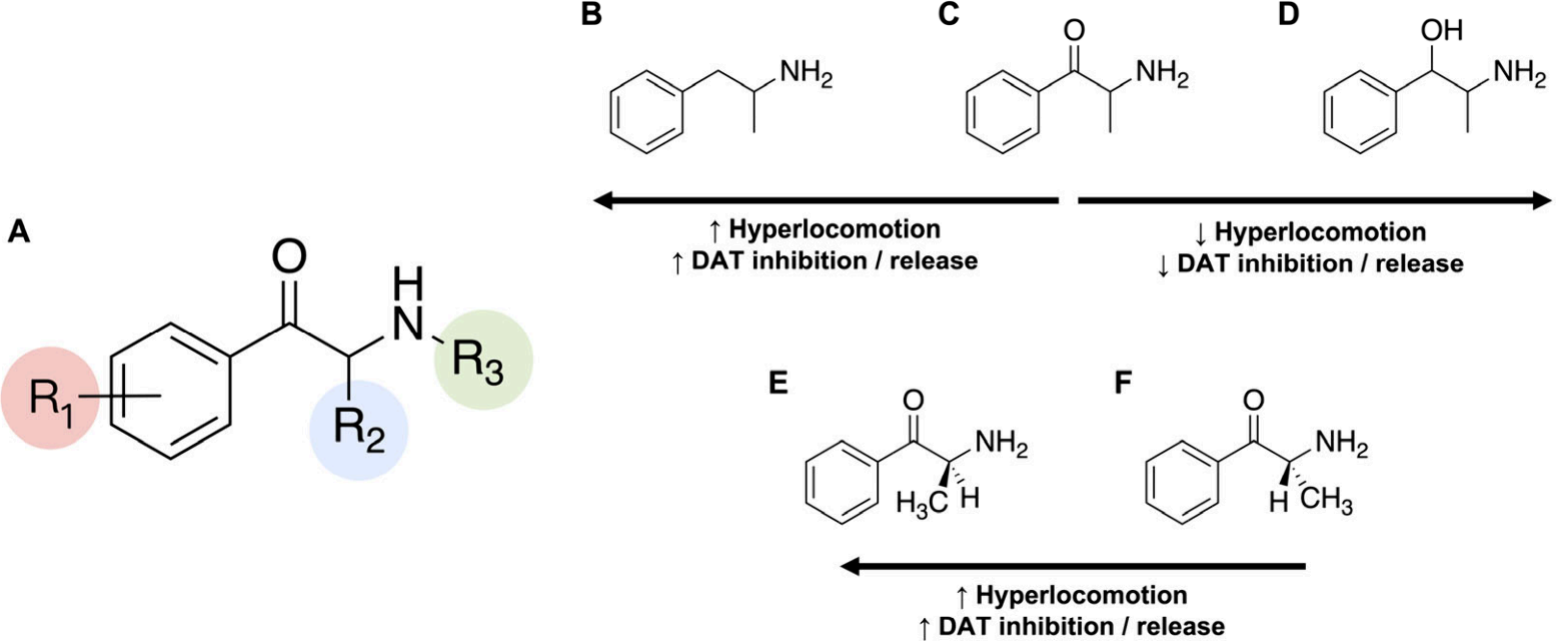
General Markush structure of substituted cathinones derivatives (A) and initial SAR insights, involving amphetamine (B), cathinone (C), cathine (D), S(−)cathinone (E), and R(+)cathinone (F).

In 1975, cathinone was identified as the active stimulant component in the *Catha edulis* shrub. Prior to this discovery, it was believed that the psychostimulant effect of the plant was mainly attributed to cathine (β-hydroxyamphetamine), first isolated from the khat plant in 1930,^127^ and later described as a central stimulant.^128^ Not that long after, it was found that cathine exhibited fewer stimulant properties than fresh khat extract, leading to the assumption that other components were in fact responsible for khat’s stimulant effects.^129^ In 1975, a United Nations laboratory isolated cathinone,^130^ and its amphetamine-like effects were soon described by various research groups.^131−134^ It was then concluded that the presence of a keto group resulted in a higher psychostimulant effect compared to a hydroxyl group. Furthermore, S(−)cathinone was found to be more potent than both (±)cathinone and R(+)cathinone.^132,135^ These early findings constituted the initial SAR insights into the cathinone structure (see Figure 3); for a comprehensive review see.^136^

Regarding differences between cathinones and amphetamines, it seems that the addition of a β-keto moiety generally decreases both *in vivo* efficacy and potency locomotor effects (see Figure 3), due to a decreased potency at inhibiting or evoking release especially at DAT, but also at NET and SERT,^137,138^ although there are some exceptions, such as 4-CMC, which showed both higher potency at inhibiting DAT and therefore higher psychostimulant effects than its amphetamine analog 4-chloro-methamphetamine (4-CMA).^137^ Moreover, an early study demonstrated that cathinone has spontaneous electrical activity through electroencephalography and motor behavior changes at much higher doses than amphetamine.^133^ Additionally, some authors have suggested that cathinones seem to have more moderate neurotoxic effects.^139,140^ For instance, Muskiewics and colleagues studied the lethality effects in mice of cathinone and its synthetic analogues MCAT, 4-MMC and MDPV in comparison to methamphetamine and MDMA, and reported that cathinone and MDPV did not produce lethality effects at any dose, MCAT only showed lethality at the highest dose tested in one subject, and 4-MMC induced lethality but at higher doses than methamphetamine and MDMA.^139^ On the other hand, *para*-halogenation of both amphetamine and methcathinone increases their neurotoxic properties due to the impairment of mitochondrial function and induction of apoptosis for both compounds, with slightly higher toxic effects of the amphetamine counterparts by means of membrane toxicity, mitochondrial membrane potential studies, intracellular ATP content, cellular oxygen consumption, mithocondrial superoxide production and mechanism of cell death studies in SH-SY5Y cells.^141^ Cathinones can be protonated in the carbonyl group due to an acidic pH in the stomach and duodenum, therefore increasing their hydrophilicity and decreasing LogD values, which results in more difficulties to penetrate the cell membrane, in comparison to amphetamines.^142^ Nonetheless, amphetamine-cathinone analogues toxicity studies should be performed in order to confirm this increased toxicity of amphetamines over cathinones is maintained for other substituents, as other studies seem to indicate that cathinones could induce seizures at lower doses than amphetamine or even - with the addition of certain substitutions in their chemical scaffold - cause more cytotoxic effects than methamphetamine.^54,133^

Synthetic cathinones can suffer modifications in the aromatic ring (i), in the length of the aliphatic side chain (ii) and in the terminal amino group (iii). Therefore, SARs aim to identify what role do these modifications in the chemical scaffold of the cathinone play in their neuropharmacological and toxicological effects. Therefore, studying the influence of these modifications on the mechanism of action, as well as in the behavioral effects and toxicity of synthetic cathinones, is an imperative and useful tool to predict the effects of NPS that will appear in the near future.

### Ring Substitutions

**Figure 4:**
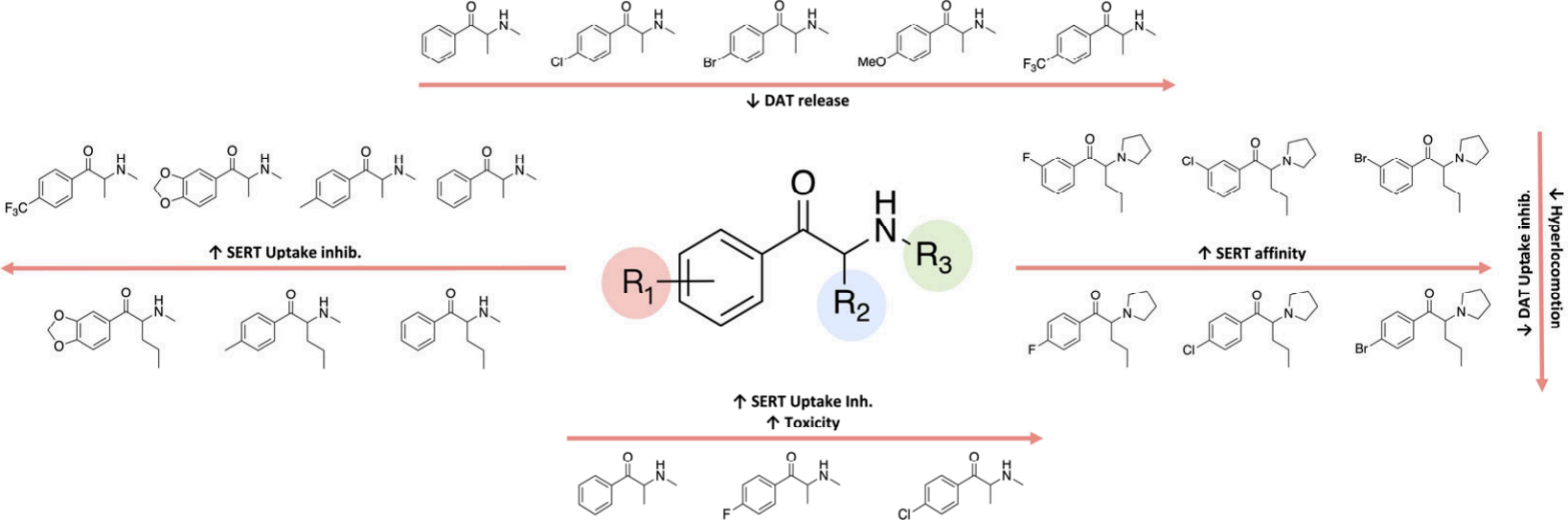
Effect of aryl substituents (R_1_) of synthetic cathinones on their mechanism of action, toxicity and *in vivo* effects.

The addition of different substituents to the aromatic region of the chemical scaffold of cathinone results in different potential to inhibit MATs. Bonano and Sakloth and colleagues^143,144^ demonstrated through Quantitative Structure–Activity Relationship (QSAR) analysis of *para*-substituted-MCAT analogs, that steric bulk of the *para* substituents plays an important role in determining the selectivity for DAT versus SERT, with larger substituents shifting the selectivity toward SERT. In accordance with this, several authors have demonstrated that the addition of both a 3,4-methylenedioxy or a *p*-methyl group to the chemical scaffold of pentedrone, MCAT and pyrovalerones leads to increased potency at inhibiting SERT, in comparison to their nonsubstituted counterparts.^36,145,146^ Dimethyl substitution at the phenyl ring of MCAT results in even higher SERT selectivity compared to the 4-methyl-substituted analogue, and at the same time in more potent inhibitors at SERT than DAT.^147^ Furthermore, addition of *p*-trifluoromethyl (*p*-CF3) to the chemical scaffold of MCAT enhances its selectivity toward the SERT compared to other substitutions.^122,148^ Moreover, *p*-CF3 in MCAT, α-PHP and synthetic cathinones structurally related to methylphenidate, resulted in cathinones with the least potency at inhibiting the DAT, in comparison to other aryl-substitutions.^73,149^ In fact, the change in properties due to presence of a *p*-CF3 substitution has been speculated to be due to its high steric hindrance,^143,144^ although other authors have also suggested that *p*-CF3 substitution can affect the pharmacokinetic and pharmacodynamic properties of synthetic cathinones.^148^ On the other hand, replacing the phenyl group of α-PBP with a thiofuran group results in α-pyrrolidinobutiothiophenone (α-PBT), which still shows psychostimulant and rewarding effects, though at high doses.^150^ Furthermore, and in contrast to its nonsubstituted analogue α-piperidinevalerophenone (α-PpVp), 3,4-propylene-2-(1-piperidinyl)valerophenone (3,4-Pr-PipVP) which contains a propylene bridge, results in equal potency at inhibiting DAT vs SERT.^151^ The introduction of bulkier substituents in different positions of the aromatic ring also plays a role in the releasing properties of synthetic cathinones. Walther and colleagues demonstrated that bulkier substituent at the *meta*- or *para*- position of MCAT results in decreased DAT release potency.^152^ The introduction of halogen atoms to the aromatic ring of synthetic cathinone is also a quite common practice within the illicit drug market,^153−156^ and it can alter the compounds’ binding affinity to MATs, as well as their behavioral effects *in vivo*. In fact, a larger volume of the halogen also results in an increased affinity for SERT, although a direct correlation between the volume of the halogen and the selectivity toward DAT is not that clear.^157−159^ Halogenation can also result in increased toxicity, in fact a bigger volume of the halogen also seems to increase toxicity.^141,159^*Para*-halogen-substituted cathinones also resulted in lower DAT/SERT inhibition ratios than their *meta*-analogs and nonsubstituted analogs, suggesting that *meta*-analogs seem to have a similar selectivity to their nonsubstituted counterpart.^157,159^ This higher serotonergic profile of *para*-substituted cathinones can also be seen when studying their releasing properties, as it has been reported that *para*-substituted MCAT derivatives are able to evoke higher 5-HT release than its *meta*-analogs.^152,160^ The increased affinity for DAT of *meta*- over *para*-substituted cathinones is also apparent *in vivo*, as *meta*-substituted cathinones have also been reported to induce higher psychostimulant effects than its *para*-analogs at the same dose.^157^ The differences observed for *para*- and *meta*- substituted cathinones to interact with DAT could be explained by the different binding mechanisms that were observed *in silico*, in which *para*-substituted-α-PVP derivatives loss the interaction with Tyr156–a critical residue for dopamine uptake^161^- and the relocation of the halogen to a neutral region of the active site, where an halogen bond cannot be stablished.^157^ On the other hand, it has also been reported that the bulkier the *para*-substituent, the more cytotoxic is the resultant cathinone.^162^ A summary of the effects that have been described for different aryl substituents on their pharmacological and toxicological effects is shown in Figure 4.

### Amine Substitutions

*N*-methylation of amphetamine has been reported to enhance its stimulant potency, and accordingly, MCAT has also been demonstrated to significantly induce higher locomotor stimulant effects in mice compared to cathinone.^163^ MCAT is classified as a DAT substrate; however, introduction of an *N*-ethyl group led to the identification of the first hybrid compound, ethcathinone (NEC), which acts as a DAT blocker but also as a 5-HT releaser.^164^ SAR studies have suggested that primary amines are optimal substrates, secondary amines are acceptable, and cathinones with tertiary amines, such as *N*-pyrrolidino-substituted cathinones, solely act as DAT and SERT blockers^35,37,165^ (for a comprehensive review, see^166^), although some α-pyrrolidinopropiophenone (α-PPP) derivatives have been reported to be partial releasing agents at NET.^167^ Sandtner and colleagues noted that interaction with certain aspartate residues (such as Asp79) is essential for the ligand to act as a substrate for DAT and SERT.^168^ Our research group recently reported that the pyrrolidine group of α-PVP does not interact with Asp79 at DAT,^157^ supporting the hypothesis that interaction with aspartate residues is crucial for substrate activity. In fact, pyrrolidinyl cathinones tend to induce mainly dopaminergic effects, therefore showing a reduction in MDMA-like effects, although substitutions on the phenyl ring seem to be able to restore these effects, albeit exclusively in compounds featuring short aliphatic side chains.^169^

Moreover, the extension of the amino group from methyl to ethyl results in a more potent DAT inhibition, whereas substituting a pyrrolidine with a diethyl and piperidine ring leads to decreased hDAT inhibition potency, which seems to be independent of other substitutions in the chemical scaffold of the cathinone.^146,170^ In correlation with these *in vitro* results, cathinones substituted with ethylamine have also been reported to have higher efficacy at inducing hyperlocomotion than those substituted with methylamine.^146^ Also, a positive correlation was observed between the hDAT/hSERT ratio and the CLogP of the amino-substituent, pointing to a heightened abuse liability associated with increased lipophilicity of the amino-substituent.^170^ Moreover, addition of a methoxy group to the terminal amine of the existing 4-MMC molecular scaffold results in the synthetic cathinone *N*-methoxymephedrone, which has been reported to have lower potency at inducing transporter-mediated release than 4-MMC.^171^

According to its increased potency at inhibiting hDAT, α-PVP and its derivatives present long lasting psychomotor effects,^157^ which could be due to recently described atypical noncompetitive pharmacology of α-PVP, which relies on a slow dissociation rate at DAT in comparison to cocaine.^172^ This slow dissociation rate was also hypothesized for MDPV.^173^ Atypical inhibitors interact with the orthosteric binding site, but elicit a conformational rearrangement, which prevents further competitive binding. The great ability of pyrrodinyl cathinones to displace and prevent further binding of [^3^H]WIN35428, a close analog of cocaine, could be of great importance for the future development of anticocaine medications, although other substitutions should be added to its chemical scaffold to increase its serotonergic potential and therefore reduce its own addictive effects.^157^ Further research should be performed to find novel molecules which are able of hindering cocaine-binding, without showing abuse potential.

**Figure 5:**
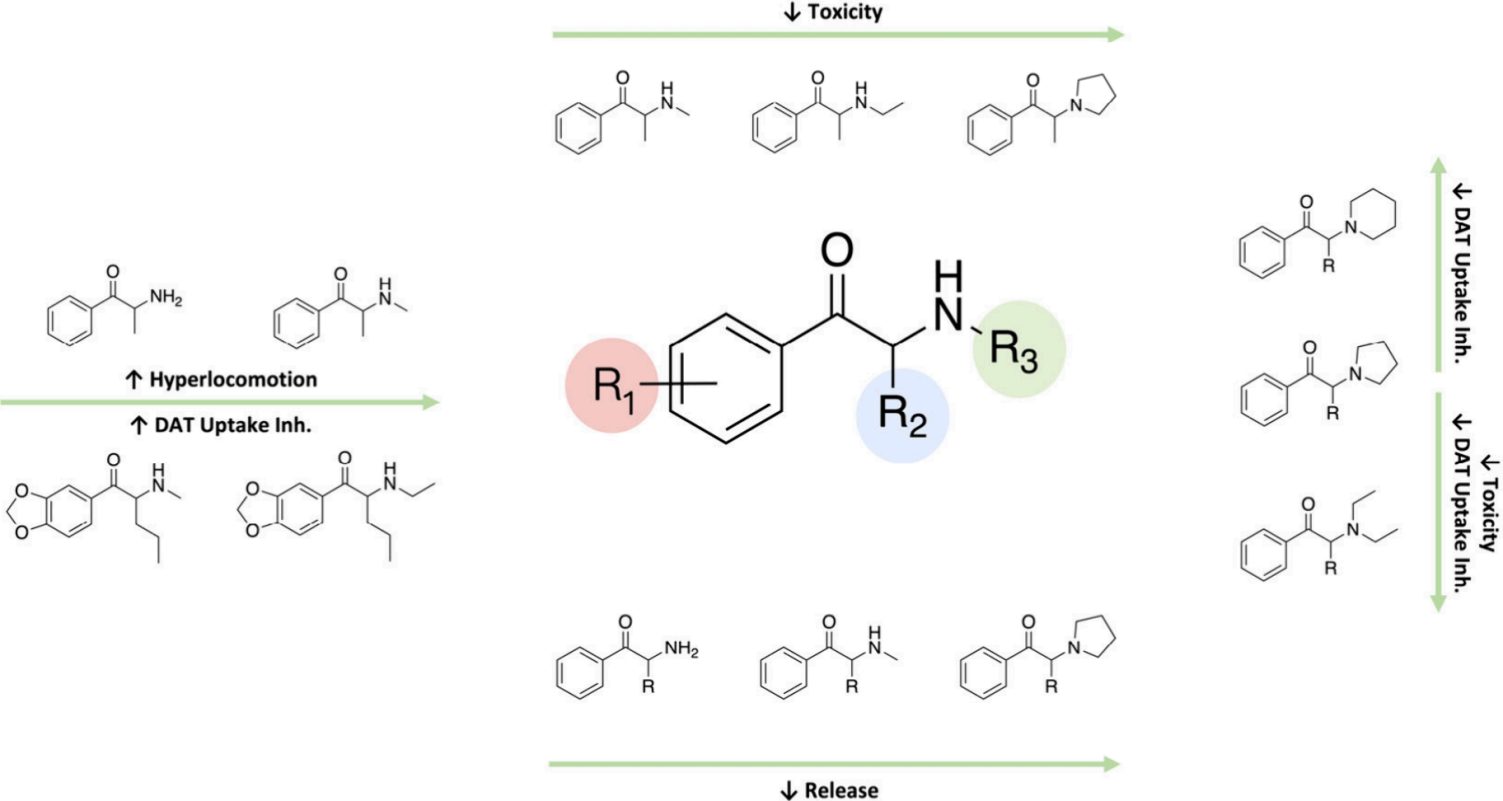
Effect of amino substituents (R_3_) of synthetic cathinones on their mechanism of action, toxicity and *in vivo* effects.

Regarding cytotoxicity, Soares et al.^162^ reported that substituting the pyrrolidine ring by a secondary amine leads to an increase of cytotoxicity, while, on the other hand, substitution of the pyrrolidine ring by a tertiary amine moiety results in a decrease of cytotoxicity. A summary of the effects that have been described for different amino terminal substitutions on their pharmacological and toxicological effects is shown in Figure 5.

### Aliphatic Side Chain Length

The addition of alkyl groups, such as methyl or ethyl, to the aliphatic side chain moiety of cathinones significantly influences their potency and pharmacokinetics, suggesting that the main metabolic pathways of α-pyrrolidinophenones significantly change depending on the alkyl chain length of the parent molecule.^174^ For instance, Matsuta et al.^175^ reported that α-PPP has a faster metabolism rate than α-PVP and α-PHP, as elongation of the aliphatic side chain resulted in a lower reduction of the keto moiety ratios and lower diastereomic ratios. Several authors have studied the effects of prolongation or shortening of the aliphatic side chain of common synthetic cathinones found in the illicit drug market, such as α-PVP, MDPV or NEPD, and in all cases coincide in an increase of the potency at inhibiting DAT when increasing the length of the aliphatic side chain from a methyl to a propyl.^36,54,176^ The effect of the length of the aliphatic side chain is also apparent *in vivo*. For instance, our group recently demonstrated that increasing the length of the aliphatic side chain of *N*-ethyl substituted cathinones from a methyl to a propyl resulted in an increased locomotor activity, but started to decrease when adding extra carbons.^54^ Some discrepancies were found with the *in vitro* results as, *N*-ethyl-heptedrone (NEHP) presented higher potency at inhibiting DAT than NEC, but induced less hyperlocomotion. Studying the kinetics and metabolism of cathinones could help understand the reasons for the discrepancies between these *in vivo* and *in vitro* findings.

On the other hand, addition of a methoxy group (−OCH3) to the α carbon of the ketone group of mephedrone results in the synthetic cathinone mexedrone, which shows weaker monoamine transporter uptake blockage and serotonin releasing properties than 4-MMC.^171^

Moreover, and in accordance with its reduced potency at inhibiting DAT when compared to α-PVP and MDPV, α-PPP and MDPP shows less potency both in the intracranial self-stimulation and in the intravenous self- administration procedure, respectively.^80,177^

Dolan and colleagues^65^ reported that methylone (methyl-) and butylone (ethyl-) fully substituted for the discriminative stimulus effect of MDMA in rats, while pentylone (propyl-) did not. A decrease in MDMA-appropiate responding was also documented for pyrrolidine-substituted cathinones as the aliphatic side chain lengthened.^169^

Nevertheless, there are also off-target effects that may be influenced by the modification of the chemical scaffold of synthetic cathinones. In fact, similarly to the DAT, Chen and Canal^178^ reported that increasing the aliphatic side chain of α-PPP to a butyl (α-PHP) results in an increase in affinity at all muscarinic receptors (MR) subtypes, though decreasing at M2Rs and M1Rs with an additional carbon.

**Figure 6:**
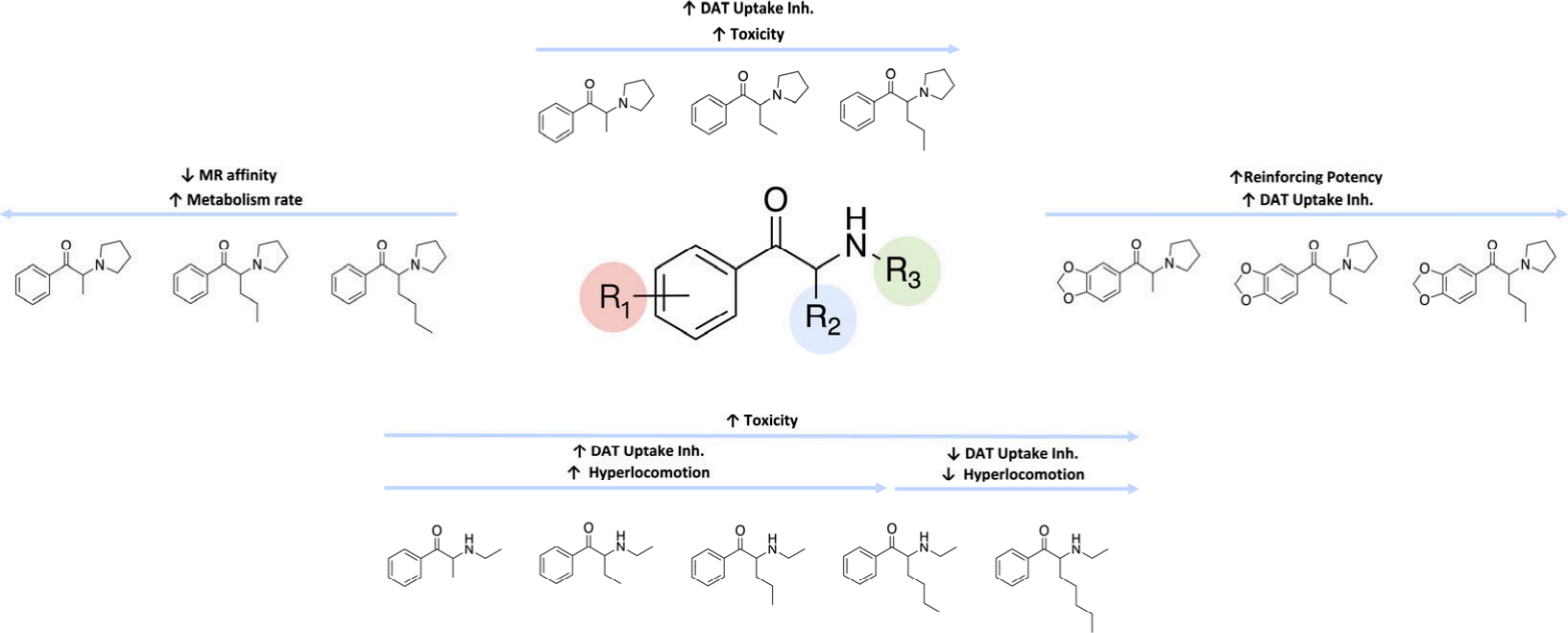
Effect of aliphatic side chain (R_2_) length of synthetic cathinones on their mechanism of action, toxicity and *in vivo* effects.

Increased alkyl chain length often correlates with enhanced lipophilicity and membrane permeability, affecting the compound’s ability to cross the blood-brain barrier.^177^ Matsunaga and colleagues^179^ demonstrated a correlation of the chain elongation in α-pyrrolidinophenones with reactive oxygen species (ROS) production. Moreover, an increase of cytotoxicity with an increase of the aliphatic side chain has also been reported for α-PVP, pentedrone and *N*-ethyl-hexedrone (NEH) analogs (α-PVP > α-pyrrolidinobutiophenone (α-PBP) > α-PPP; pentedrone > buphedrone > MCAT; NEHP > NEH > NEPD > *N*-ethyl-buphedrone (NEB)) either through the 3-(4,5-dimethylthiazol-2-yl)-2,5-diphenyl tetrazolium (MTT) or the 2-(2-methoxy-4-nitrophenyl)-3-(4-nitrophenyl)-5-(2,4-disulfophenyl)-2H-tetrazolium, monosodium salt (WST-8) assay.^54,162^ A summary of the effects that have been described for different length of the aliphatic side chain on their pharmacological and toxicological effects is shown in Figure 6.

## Concluding Remarks

In this article we have reviewed the significant impact of chemical modifications in the scaffold of synthetic cathinones on their mechanisms of action, behavioral effects, and neurotoxic profiles. Ring substitutions, particularly the addition of bulkier groups or halogens to the aromatic region, play a pivotal role in modulating the selectivity and potency of these compounds toward MATs. Larger substituents tend to enhance selectivity for SERT over DAT, while also potentially increasing toxicity. Amine substitutions, such as *N*-methylation or the incorporation of pyrrolidine rings, markedly influence the stimulant potency and substrate activity at MATs. The introduction of bulkier amine groups seems to decrease cytotoxicity, though it may also heighten abuse potential and psychostimulant effects. Additionally, variations in the aliphatic side chain length significantly affect the pharmacokinetics, DAT inhibition potency, and behavioral outcomes of synthetic cathinones, generally increasing with the length of the aliphatic side chain. However, in order to fully elucidate the complexities of these modifications and their implications for human health and safety, further research focusing on SAR of synthetic cathinones is needed.

Analyzing the SAR of synthetic cathinones is pivotal for multiple reasons. It provides crucial insights into how the structural characteristics of these compounds influence their biological effects, therefore being instrumental in predicting potential toxicities and side effects associated with structural modifications. Additionally, SAR knowledge may be vital for regulatory compliance, assisting authorities in promptly categorizing and regulating this new class of compounds. Forensic identification techniques also benefit from SAR understanding, facilitating accurate detection. The information derived contributes to informed drug policies, addresses public health concerns, and prevents the exploitation of designer drugs. Moreover, SAR studies may uncover potential medical applications, opening avenues for the development of lead compounds for specific neurological or psychiatric conditions. In essence, exploring the SAR of synthetic cathinones involves a comprehensive approach that can contribute to toxicity assessment, regulatory compliance, forensic identification, and potential therapeutic applications.

## Abbreviations

NPSs: New Psychoactive Substances
EU-EWS: European Union – Early Warning System
UNODC: United Nations Office on Drugs and Crime
EWA: Early Warning Advisory
3-CMC: 3-chloromethcathinone
3-MMC: 3-methylmethcathinone
4-CMC: 4-chloromethcathinone
MDMA: 3,4-methylenedioxymethamphetamine
MCAT: methcathinone
4-MMC: 4-methylmethcathinone
MDPV: 3,4-methylenedioxypyrovalerone
SAR: Structure–Activity Relationship
MAT: Monoamine Transporter
SLC6: Solute carrier 6
DAT: Dopamine Transporter
SERT: Serotonin Transporter
NET: Norepinephrine Transporter
DAT: Dopamine
NE: Norepinephrine
5-HT: Serotonin
VMAT: Vesicular monoamine transporter
HEK293: Human embryonic kidney cells
3-FMC: 3-fluoromethcathinone
4-MEC: 4-methylethcathinone
α-PVP: α-pyrrolidinopentiophenone
α-PHP: α-pyrrolidinohexiophenone
α-POP: α-pyrrolidinooctanophenone
NEPD: *N*-etlyl-pentedrone
VTA: Ventral tegmental area
NAcc: Nucleus accumbens
VS: Ventral striatum
DS: Dorsal striatum
ICSS: Intracranial self-stimulation
GC-MS/MS: Gas chromatography coupled with (tandem) mass spectrometry
HPLC-MS/MS: Liquid chromatography with (tandem) mass spectrometry
IMS: Ion mobility spectrometry
CE: Capillary electrophoresis
4-MeAP: 4-methyl-α-ethylaminopentiophenone
4-MNEB: 4-methyl-*N*-ethylbuphedrone
4-MDMP: 4′-methyl-*N*,*N*-dimethylpentedrone
EMA: European Medicines Agency
FDA: Food and Drug Administration
4-CMA: 4-chloro-methamphetamine
QSAR: Quantitative Structure–Activity Relationship
*p*-CF3: *p*-trifluoromethyl
α-PBT: α-pyrrolidinobutiothiophenone
α-PpVp: α-piperidinevalerophenone
3,4-Pr-PipVP: 3,4-propylene-2-(1-piperidinyl)valerophenone
NEC: ethcathinone
α-PPP: α-pyrrolidinopropiophenone
NEHP: *N*-ethyl-heptedrone
-OCH3: Methoxy group
MRs: Muscarinic receptors
ROS: Reactive oxygen species
NEH: *N*-ethyl-hexedrone
α-PBP: α-pyrrolidinobutiophenone
NEB: *N*-ethyl-buphedrone
MTT: 3-(4,5-dimethylthiazol-2-yl)-2,5-diphenyl tetrazolium
WST-8: 2-(2-methoxy-4-nitrophenyl)-3-(4-nitrophenyl)-5-(2,4-disulfophenyl)-2*H*-tetrazolium, monosodium salt
